# Supplementary material for: Multimodal collective swimming of magnetically articulated modular nanocomposite robots
Source: Nat Commun. 2022 Nov 8;13:6750. doi: 10.1038/s41467-022-34430-2 (PMC9643480; doi:10.1038/s41467-022-34430-2)
Supplement: Supplementary file 1 — Supplementary Information [file 41467_2022_34430_MOESM1_ESM.pdf]

Supplementary Information:

Multimodal Collective Swimming of Magnetically Articulated Modular Nanocomposite Robots

Sukyoung Won[1,2,†], Hee Eun Lee[3,†], Young Shik Cho[4], Kijun Yang[1,2], Jeong Eun Park[1,2], Seung Jae Yang[5,*], Jeong Jae Wie[6,7,8,9,*]

[1]The Research Institute of Industrial Science, Hanyang University, Seoul, 04763, Republic of Korea

[2]Program in Environmental and Polymer Engineering, Inha University, Incheon, 22212, Republic of Korea

[3]Green Product Solution Center, SK Innovation, Daejeon, 34124, Republic of Korea

[4]Department of Materials Science & Engineering and Research Institute of Advanced Materials (RIAM), Seoul National University, Seoul, 08826, Republic of Korea

[5]Advanced Nanohybrids Laboratory, Department of Chemistry and Chemical Engineering, Education and Research Center for Smart Energy and Materials, Inha University, Incheon, 22212, Republic of Korea

[6]Department of Organic and Nano Engineering, Hanyang University, Seoul, 04763, Republic of Korea

[7]Human-Tech Convergence Program, Hanyang University, Seoul, 04763, Republic of Korea

[8]Department of Chemical Engineering, Hanyang University, Seoul, 04763, Republic of Korea

[9]Institute of Nano Science and Technology, Hanyang University, Seoul, 04763, Republic of Korea

Supplementary Table 1. Dimensions, body lengths, body masses, and saturation magnetization (M_s) values of carbon nanotube yarn (CNTY) robots. The vol.% notation refers to the concentration of iron particles dispersed in the PDMS prepolymer.

Aspect ratio/ particle concentration	Diameter (mm)	Body length (mm)	Body mass (mg)	M_s (emu g^{-1})
AR-2.5/10-vol.%	0.37	0.92	0.11	69
AR-6/10-vol.%	0.37	2.2	0.27	69
AR-13/10-vol.%	0.37	4.8	0.59	69
AR-2.5/20-vol.%	0.38	0.96	0.18	118
AR-6/20-vol.%	0.38	2.3	0.43	118
AR-13/20-vol.%	0.38	5.0	0.92	118
AR-2.5/30-vol.%	0.46	1.2	0.26	152
AR-6/30-vol.%	0.46	2.8	0.64	152
AR-13/30-vol.%	0.46	6.0	1.38	152

Supplementary Table 2. Swimming speeds of living swimmers.

**Living swimmer**	**Swimming type**	**Body length (mm)**	**Swimming speed (BL s^{-1}; BL: body length)**	**Supplementary reference**
Water strider (*Gerridae*)	Above water	10	10.0	1
Mallard (*Anas platyrhynchos*)	Above water	330	2.2	2
Basilisk lizard (*Basiliscus plumifron)*	Above water	600	2.7	3
Yellow-bellied sea snake (*Pelamis platura*)	Above water	705	0.75	4
Ciliate *(Paramecium)*	Underwater	0.35	5.7	5
Roundworm *(Caenorhabditis elegans)*	Underwater	1	0.4	6
Copepod (*Metridia pacifica*)	Underwater	2.5	7.0	7
Brown shrimp (*Crangon crangon*)	Underwater	40	18.8	8
Ghost crab (Ocypode quadrata)	Underwater	76	11	9
Bottlenose dolphin (*Tursiops gilli*)	Underwater	1910	4.3	10
Toothed whale (*odontocete cetaceans*)	Underwater	4550	1.4	11

Supplementary Table 3. Swimming speeds of miniaturized artificial magnetic swimmers in single magnetic robot systems.

Artificial magnetic swimmer	Swimming type	Body length (mm)	Aspect ratio (body length per body width)	Maximum swimming speed (BL s^{-1}; BL: body length)	Supplementary reference
Present study (musculoskeletal system-mimetic robot)	Above water	1.2	2.5	180	-
	Above water	2.8	6	66	-
	Above water	6	13	40	-
Elastically linked ferromagnet	Above water	0.068	1.4	1.4	12
Pentagram-shaped robot	Above water	0.45	1	2.2	13
Helical robot	Above water	0.6	2	4	14
Spermatozoid-like robot	Above water	10	3.3	1.1	15
Janus dimer particle	Underwater	0.01	2	13.3	16
Magnetotactic bacteria-mimetic robot	Underwater	0.05	1	3.2	17
Cuboid-shaped robot	Underwater	0.1	5	2.8	18
Helical robot	Underwater	0.6	2	56	19
Rectangular-shaped robot	Underwater	0.8	2	17	20
Spherical robot	Underwater	0.977	1	7.23	21
Helical robot	Underwater	2	2	0.55	22
Cross-shaped robot	Underwater	7	1	2	23

Supplementary Figures

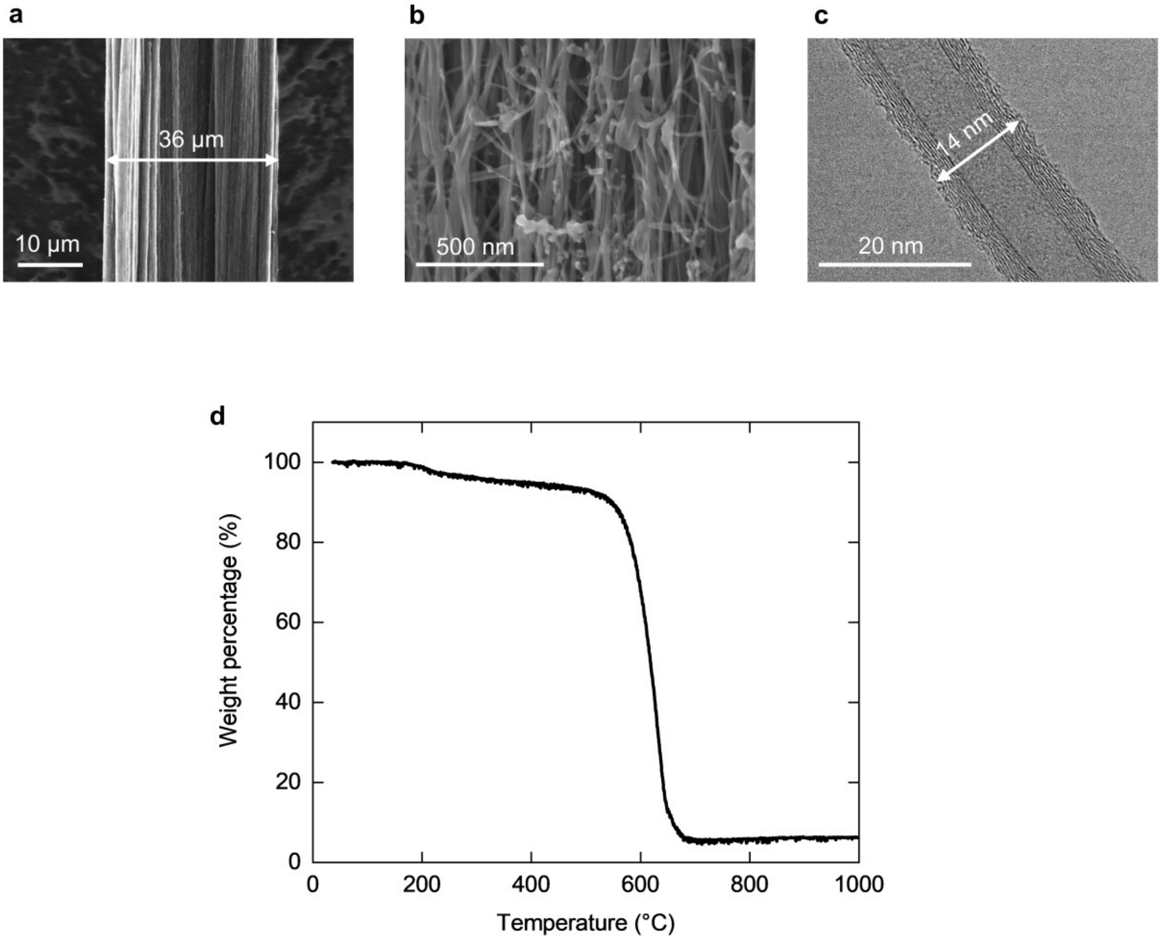

Supplementary Fig. 1 Physical properties of multi-walled carbon nanotube (MWNT). Scattering electron microscope (SEM) images of 36-μm-thick single MWNT fiber in (a) low-magnification (top-down view) and (b) high-magnification (radial section view). (c) Transmission electron microscope (TEM) image (top-down view) of a ten-walled 14 nm-thick MWNT composing of the MWNT fiber. (d) Obtained the MWNT fiber with high purity, confirmed by thermogravimetric analyzer (TGA) with a heating rate of 10 °C min^{-1} in an air atmosphere. The weight loss of the MWNT fiber was 86% and residual weight was 6%.

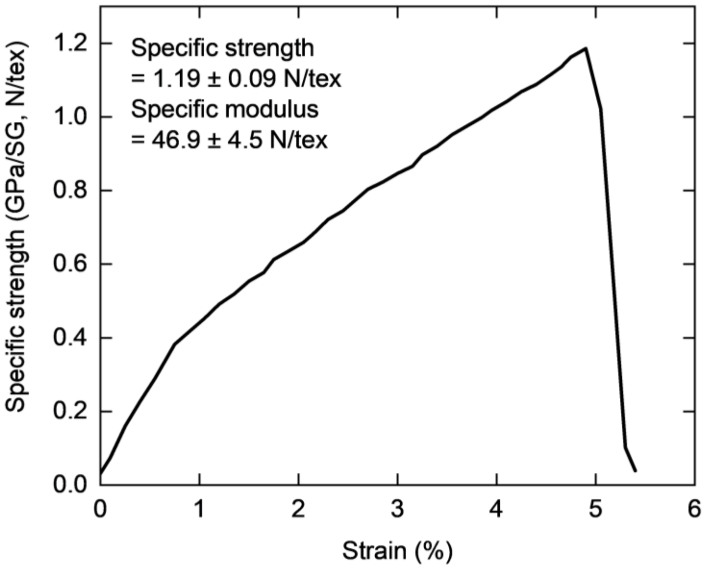

Supplementary Fig. 2 Stress-strain curve of the single MWNT fiber. Tensile test was performed at 3 mm min^{-1} and gauge length was 1 cm. Specific strength and specific modulus reached up to 1.19 ± 0.09 N tex^{-1} (equivalent to 1.19 GPa/SG) and 46.9 ± 4.5 N tex^{-1}, respectively (n= 10). SG denotes specific gravity, the density of the materials divided by the density of water. The bulk density of MWNT fiber was 2.6 g cm^{-3}.

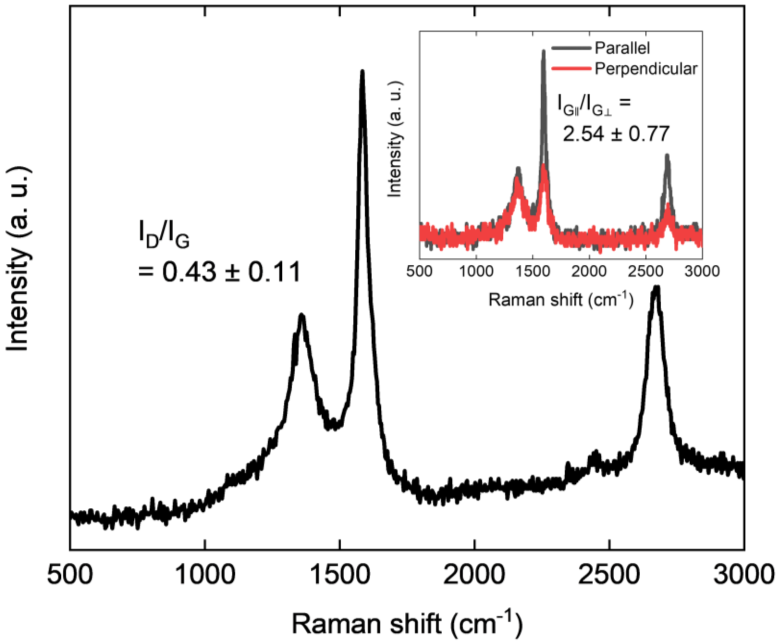

Supplementary Fig. 3 Raman spectra (inset: polarized Raman spectra) of synthesized MWNT. The intensity ratio of the D-band to G-band (I_D/I_G) in the Raman spectra, which states the crystallinity of MWNT, was approximately 0.43 ± 0.11 (*n*= 5). The G peak intensity ratio ($I_{G\parallel}/I_{G\perp}$) of the parallel direction to the perpendicular direction along to the CNTY axis in the polarized Raman spectrum, which states the orientation of MWNT, was 2.54 ± 0.77 (*n*= 5). For lightweight nanoporous structure, high densification was intentionally restrained in the MWNT fiber, confirmed by the crystallinity and orientation[24,25].

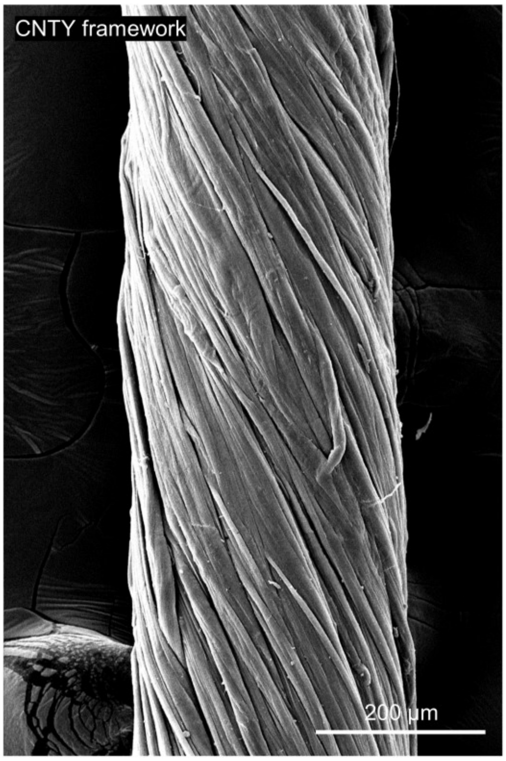

Supplementary Fig. 4 CNTY framework. 100 MWNT fibers were twisted to form the 340-μm-thick CNTY.

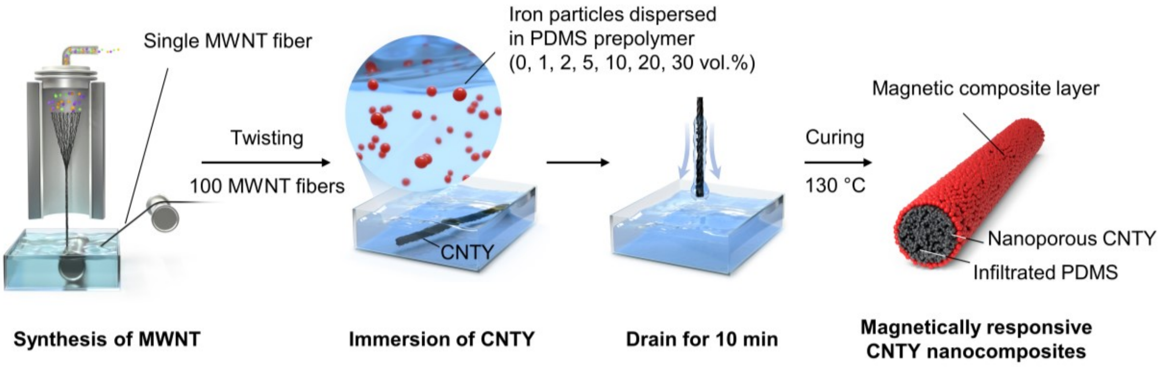

Supplementary Fig. 5 Schematic illustration of the preparation of biomimetic hierarchical nano/microstructures by floating catalyst chemical vapor deposition and dip-coating methods.

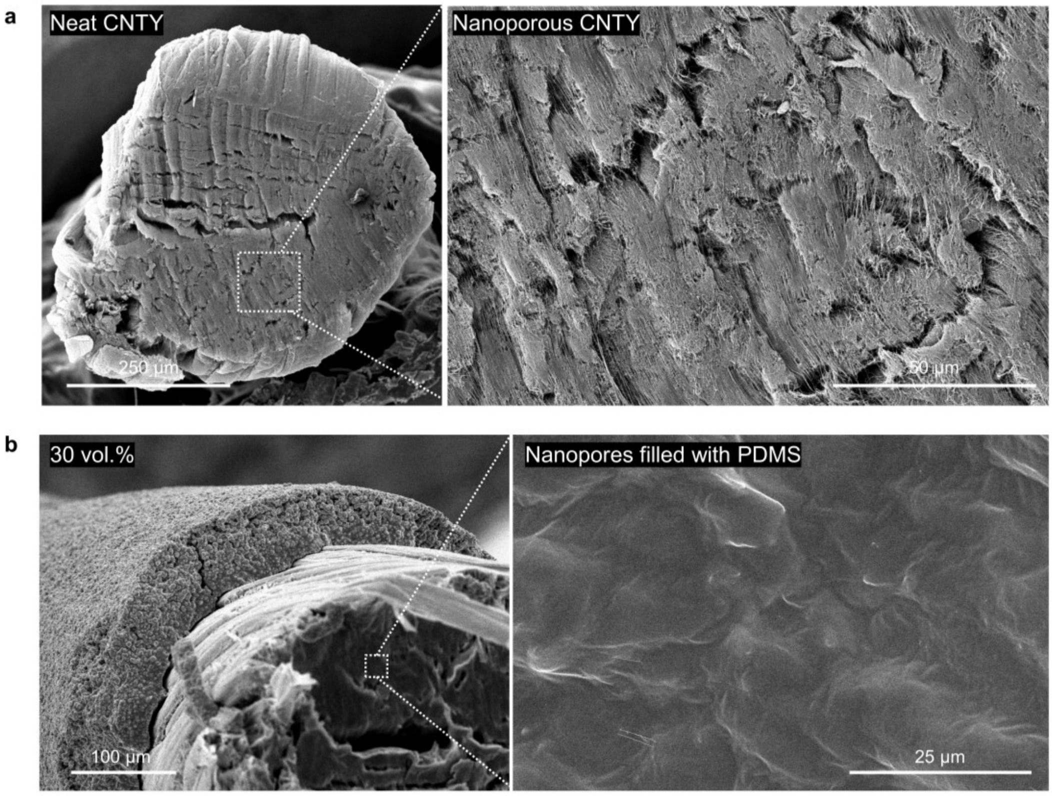

Supplementary Fig. 6 Cross-sectional SEM images showing nanopores in the CNTY framework. (a) Porous CNTY. (b) CNTY framework containing PDMS that infiltrated into its nanopores by capillary action during the dip-coating process. Micron-scale iron particles were excluded by the nanopores. The 30 vol.% notation refers to the concentration of iron particles dispersed in the PDMS prepolymer.

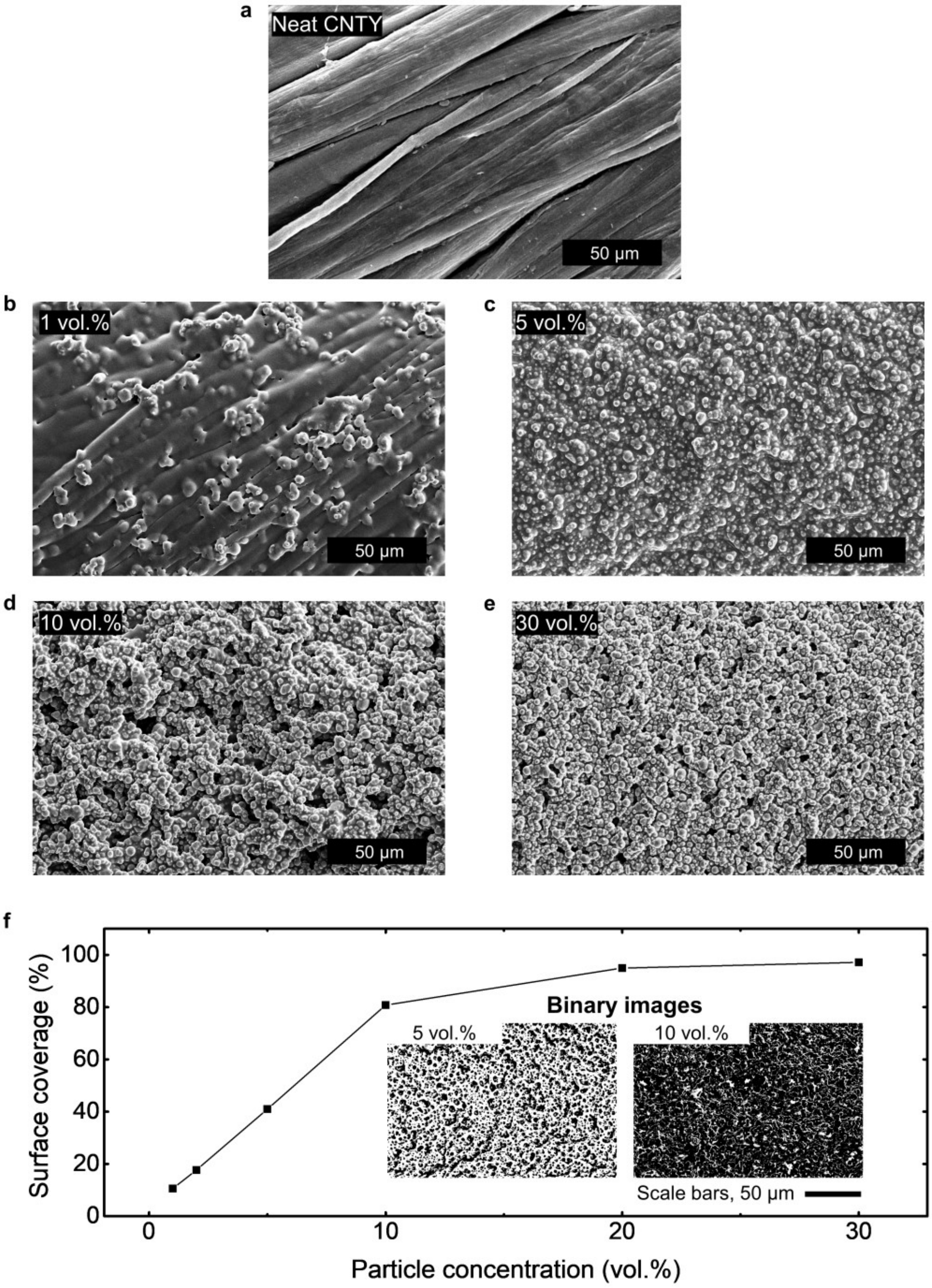

Supplementary Fig. 7 Surface analysis of the CNTY framework coated with a magnetic composite layer based on SEM images in the top-down view. (a) High-magnification image of neat CNTY surface. (b)–(e) The CNTY surface coated with the magnetic composite layer containing iron particle concentrations of (b) 1, (c) 5, (d) 10, and (e) 30 vol.% dispersed in the PDMS prepolymer. (f) Surface coverage of the magnetic particles estimated from binary images; the inset images show representative binary images.

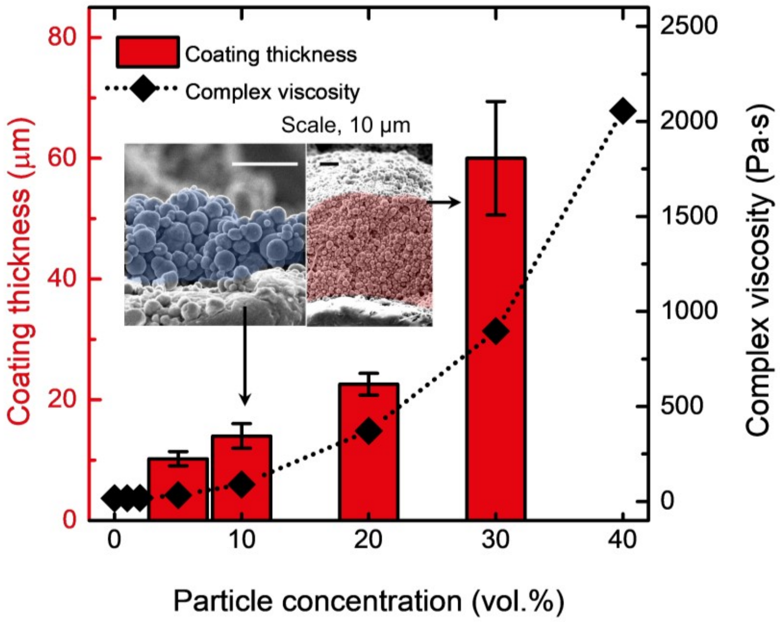

Supplementary Fig. 8 Dependence of coating thickness of magnetic composite layer on complex viscosity of PDMS prepolymer composite. The complex viscosities were calculated at an angular frequency of 3 rad s^{-1}. The cross-sectional micrographs in the inset show the coating layer of the PDMS–iron-particle composite layer with the condition of 10 vol.% (left) and 30 vol.% (right) iron particles. All error bars represent the standard deviation ($n = 3$). At a particle concentration of 40 vol.%, uniform dip-coating of the magnetic composite layer on CNTY was hindered due to the high complex viscosity.

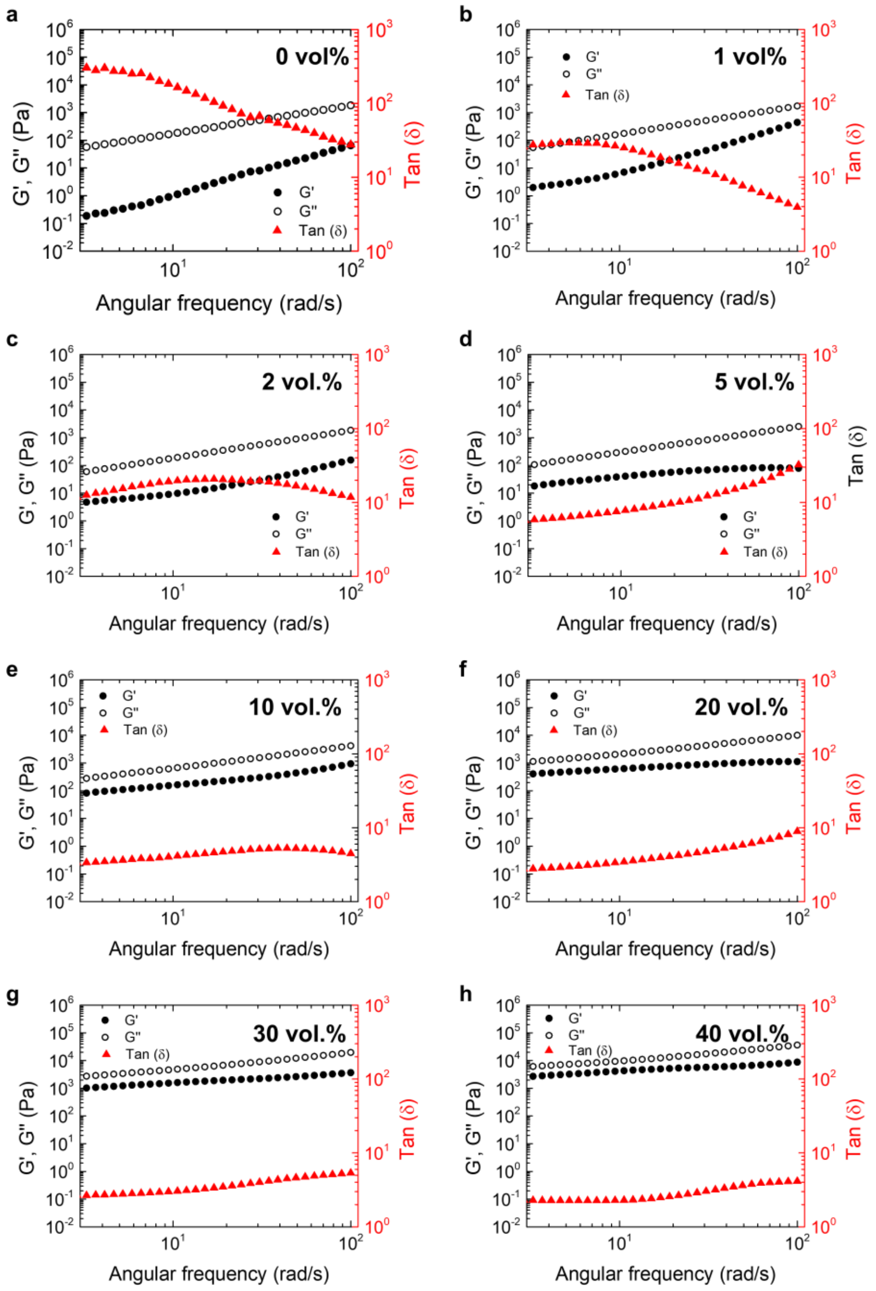

Supplementary Fig. 9 Viscous flow behavior of PDMS–magnetic-particle mixtures. Storage modulus (G'; filled circles), loss modulus (G''; open circles), and tan(δ) (red triangles) were measured using a rheometer. Iron particle concentration in the PDMS prepolymer is (a) 0, (b) 1, (c) 2, (d) 5, (e) 10, (f) 20, (g) 30, and (h) 40 vol.%.

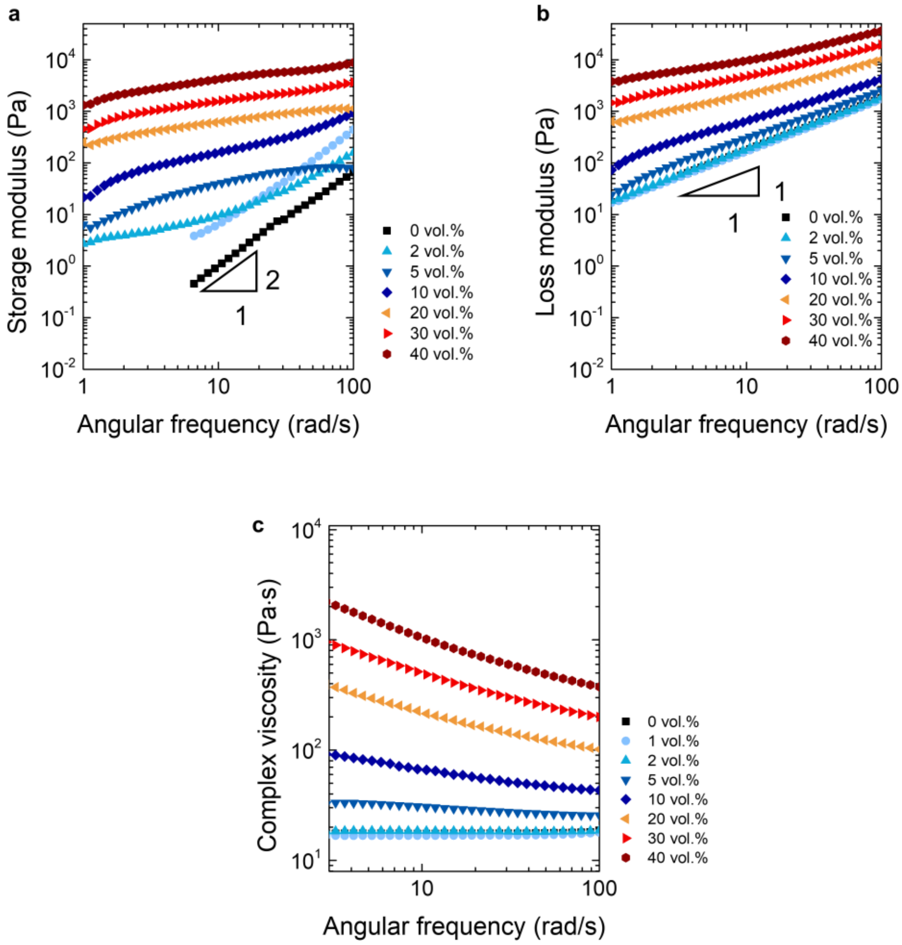

Supplementary Fig. 10 Viscosity measurements. (a) Storage moduli, (b) loss moduli, and (c) the resultant complex viscosities of the PDMS–magnetic-particle mixtures.

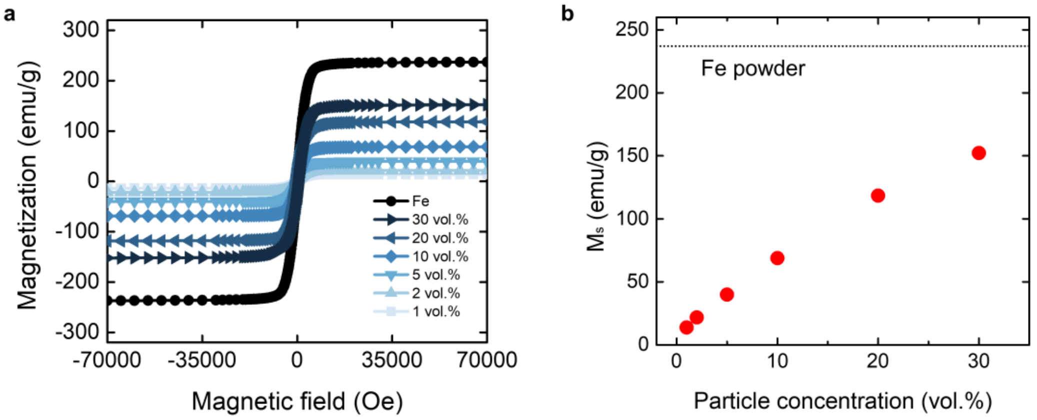

Supplementary Fig. 11 Magnetization properties of the ternary nanocomposite. (a) Magnetization of bulk iron particles and the ternary nanocomposites measured using a vibrating-sample magnetometer. (b) M_s of bulk iron particles and the ternary nanocomposites, measured at 70 kOe. The M_s of bulk powder of iron particles was estimated to be 237 emu g^{-1}, and those of the ternary nanocomposites were found to be 14, 22, 40, 69, 118, and 152 emu g^{-1} at magnetic particle concentrations of 1, 2, 5, 10, 20, and 30 vol.% in the PDMS prepolymer, respectively.

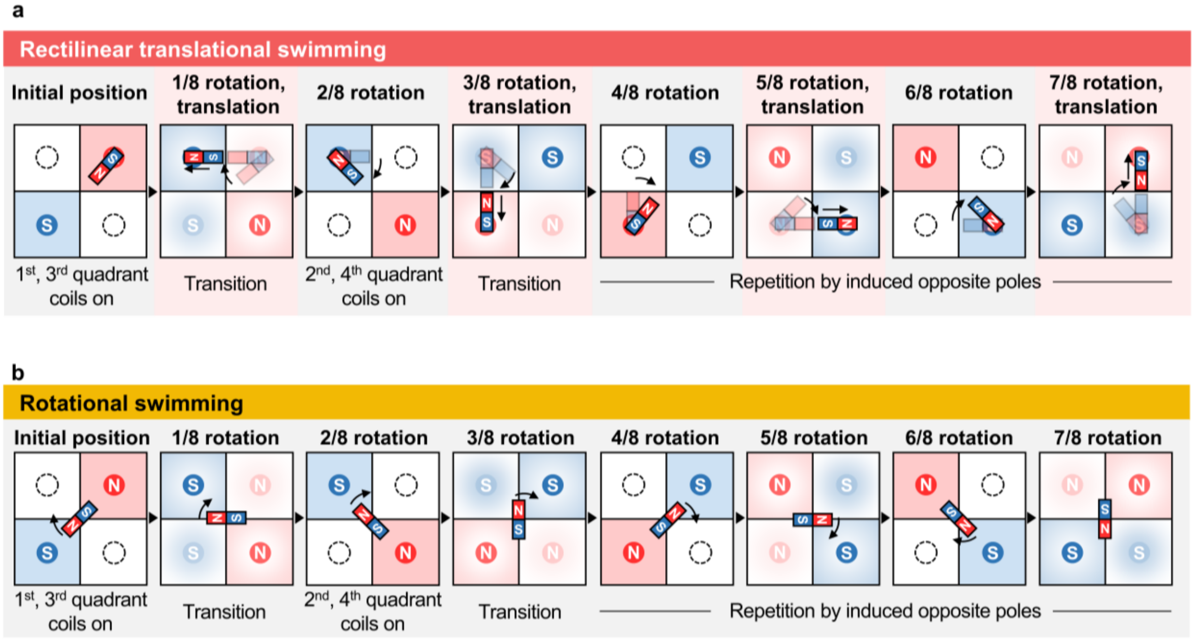

Supplementary Fig. 12 Bimodal above-water swimming of CNTY robots. An inductive quadrupolar electromagnetic field enables the CNTY robot to swim in the rectilinear translational swimming or rotational swimming modes. To generate one rotation of the pulsed electromagnetic field, the north pole was generated at the 1st, 4th, 3rd, and 2nd quadrants in sequence, while the pair coil in the 3rd, 2nd, 1st, and 4th quadrants sequentially generated the south pole. (a) Rectilinear translational swimming of a CNTY robot at a low magnetic frequency. Rectilinear translation was achieved by the gradient of the magnetic field magnitude generated in the transition state of the switching on/off states of the coil pairs. Vertex rotation was realized by the magnetic torque when the subsequent coil pair was switched on. The magnetic robot can initiate swimming at the 1st or 3rd quadrant. (b) Rotational swimming of the CNTY robot at a high magnetic frequency.

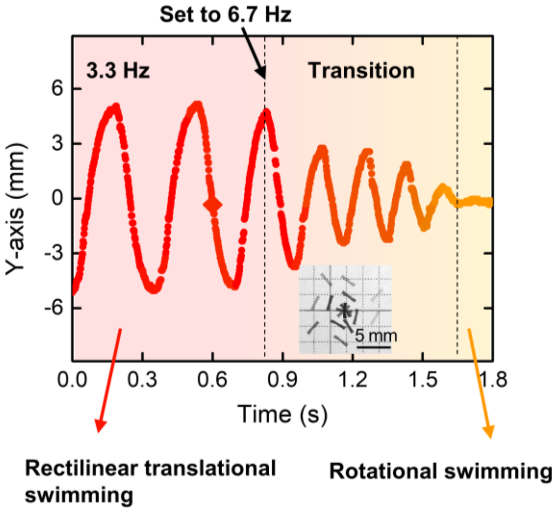

Supplementary Fig. 13 Transition state of swimming modes. Transition from rectilinear translational swimming to rotational swimming of the AR-6/10-vol.% CNTY robot upon shifting of the magnetic frequency from 3.3 to 6.7 Hz.

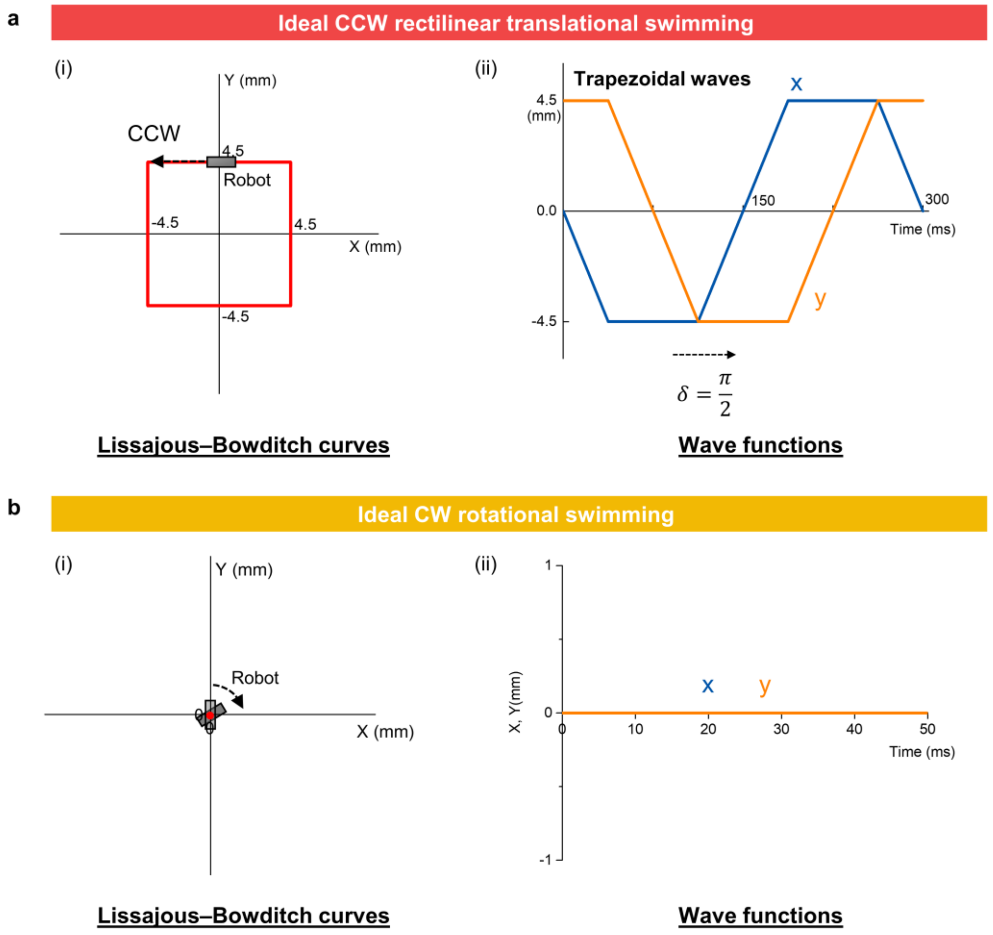

Supplementary Fig. 14 Ideal bimodal swimming of CNTY robots actuated by a quadrupolar electromagnetic field. (a) Ideal counterclockwise (CCW) rectilinear translational swimming represented by a (i) Lissajous–Bowditch curve and (ii) trapezoidal waves. The phase difference, δ, between the x-axis and y-axis coordinates of the robots shows a positive value of 1.57 rad. (b) Ideal clockwise (CW) rotational swimming represented by a (i) Lissajous–Bowditch curve and (ii) linear profiles. The phase difference disappears in rotational swimming.

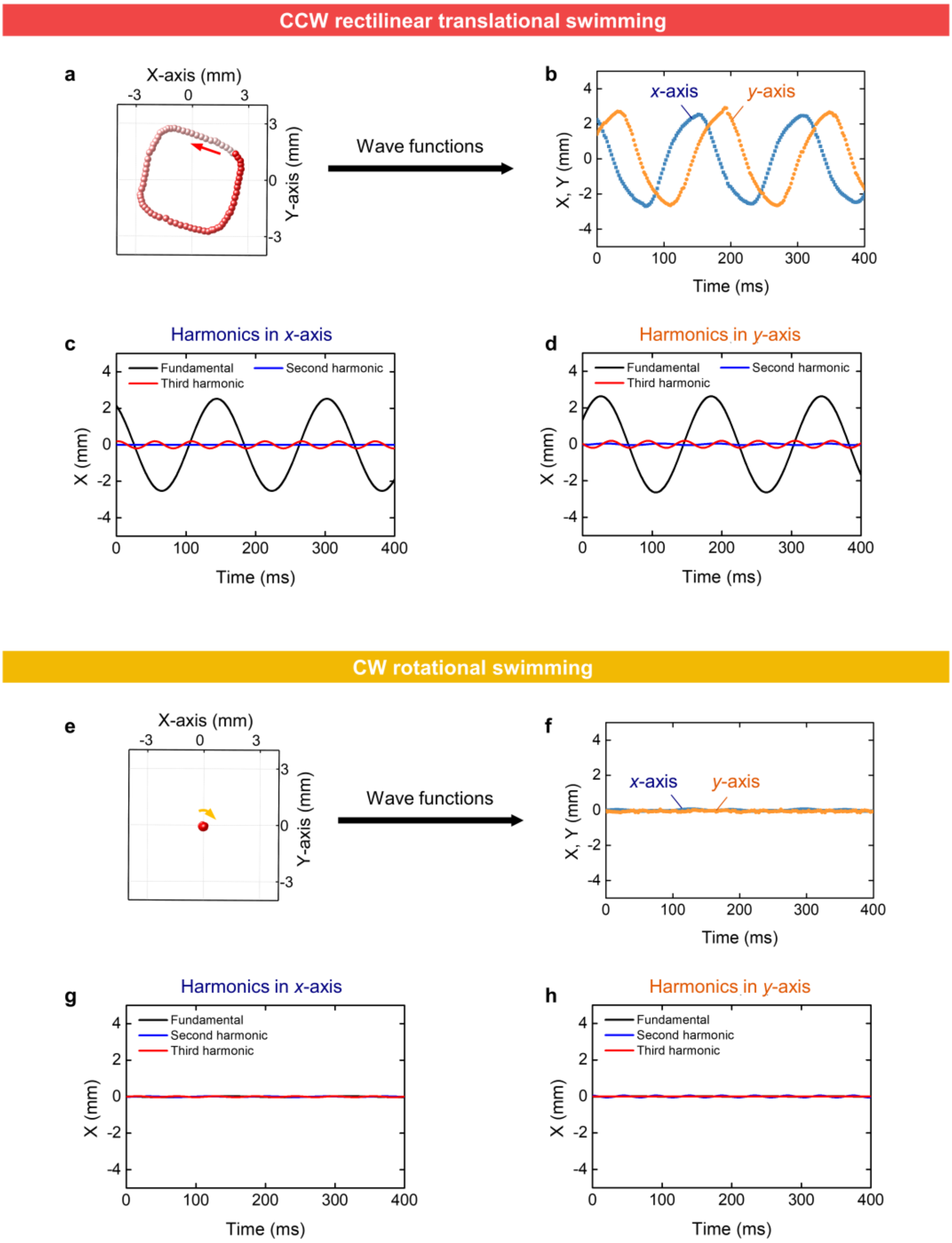

Supplementary Fig. 15 Harmonics in bimodal swimming fitted to Fourier series. (a)–(d) Representative rectilinear translational swimming of AR-2.5/20-vol.% at a magnetic frequency of 6.7 Hz. (a) Swimming trajectory of the robot and (b) sinusoidal waves of the *x*-axis and *y*-axis coordinates. (c) *x*-axis and (d) *y*-axis coordinates fitted to Fourier series. (e)–(h) Representative rotational swimming of AR-2.5/10-vol.% at a magnetic frequency of 6.7 Hz. (e) Swimming trajectory of the robot and (f) sinusoidal waves of the *x*-axis and *y*-axis coordinates. (g) *x*-axis and (h) *y*-axis coordinates fitted to Fourier series.

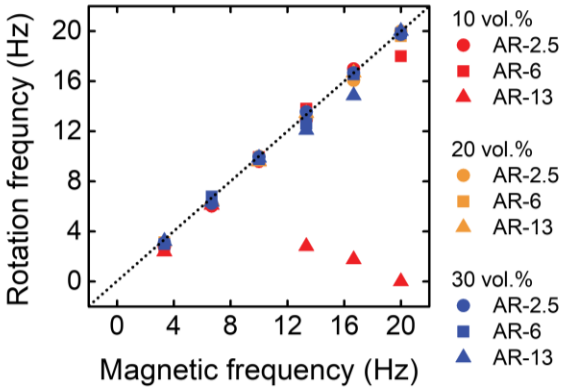

Supplementary Fig. 16 Rotation frequency of CNTY robots. The rotation frequency corresponds to the magnetic frequency of the pulsed electromagnetic field. The AR-13/10-vol.% robot, which showed results that were an exception to these data, exhibited a step-out frequency of 10 Hz, indicating that it could not perform magnetic rotation at magnetic frequencies of 13.3 and 16.7 Hz. The AR-13/10-vol.% robot eventually came to a halt at a magnetic frequency of 20 Hz.

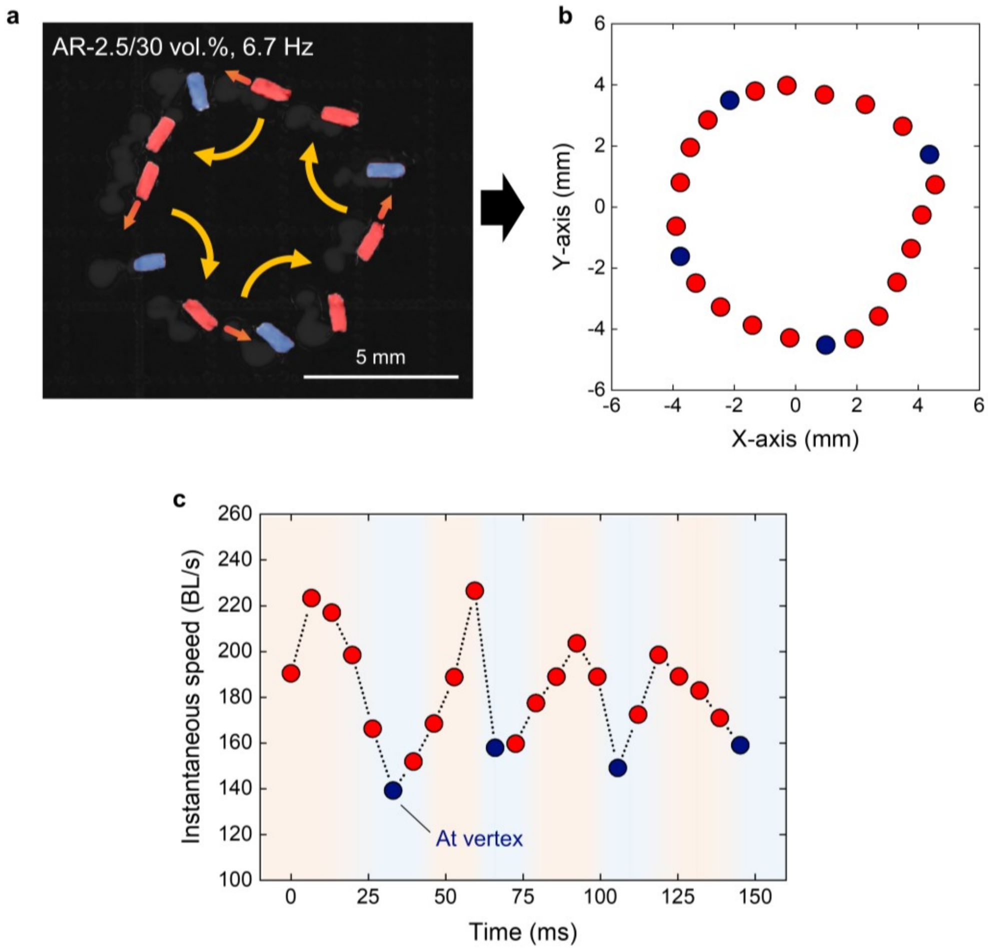

Supplementary Fig. 17 Speed analysis of rectilinear translational swimming. (a) Overlaid image of the center of mass of AR-2.5/30-vol.% CNTY robot at 6.7 Hz. (b) Corresponding trajectory of (a). The robot was shown in red to distinguish the shadows reflected on water. The blue colors indicate position at the vertex in the trajectory. (c) Instantaneous speed of the CNTY robot. The CNTY robot showed fluctuation of instantaneous velocity since the pulsed electromagnetic field induced rectilinear translational motion and rotational motion for the robots (see Supplementary Fig. 12). Maximum instantaneous speed was 227 BL s^{-1}. We calculated an average swimming speed using the total swimming distance of CNTY robots divided by the swimming period. The average swimming speed of the AR-2.5/30-vol.% CNTY robot was 180 ± 24 BL s^{-1}.

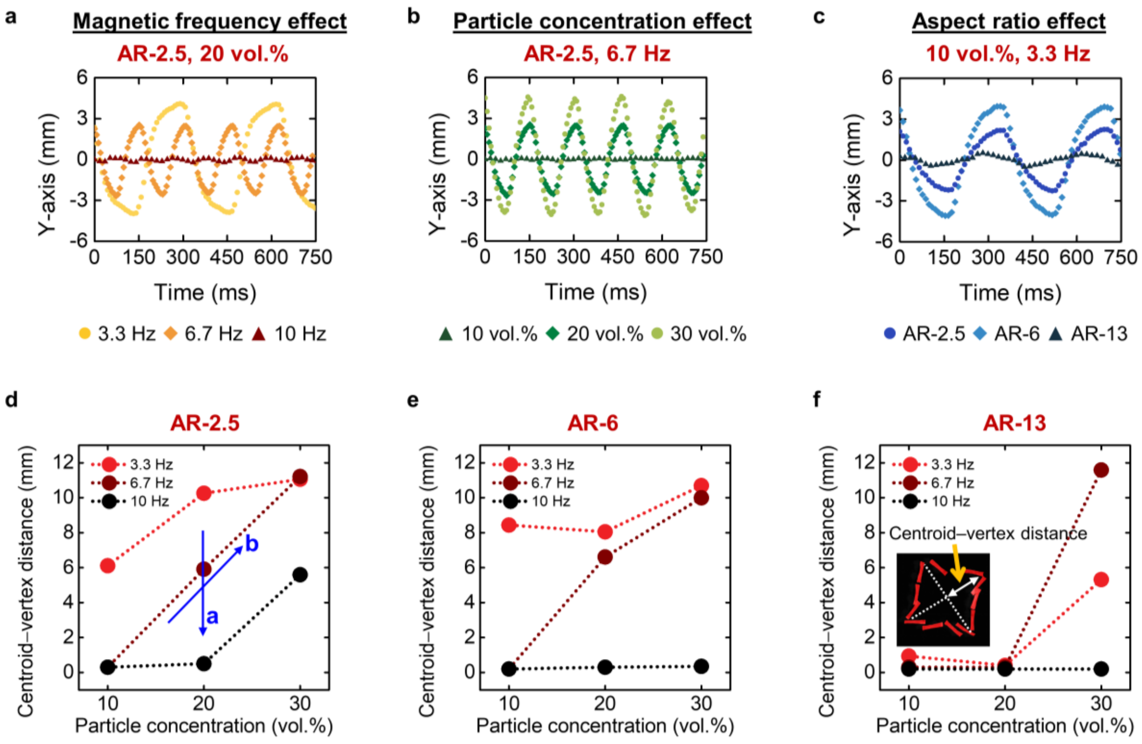

Supplementary Fig. 18 Centroid–vertex distance analysis. Representative effects of (a) magnetic frequency, (b) particle concentration, and (c) aspect ratio (AR) on bimodal swimming. Measured centroid–vertex distances in the (d) AR-2.5-, (e) AR-6-, and (f) AR-13-based robots as a function of particle concentration. The AR affects the inertial force due to M_s and mass, showing a miniscule orbit (AR-13/10-vol.%, 3.3 Hz) and complex swimming behavior.

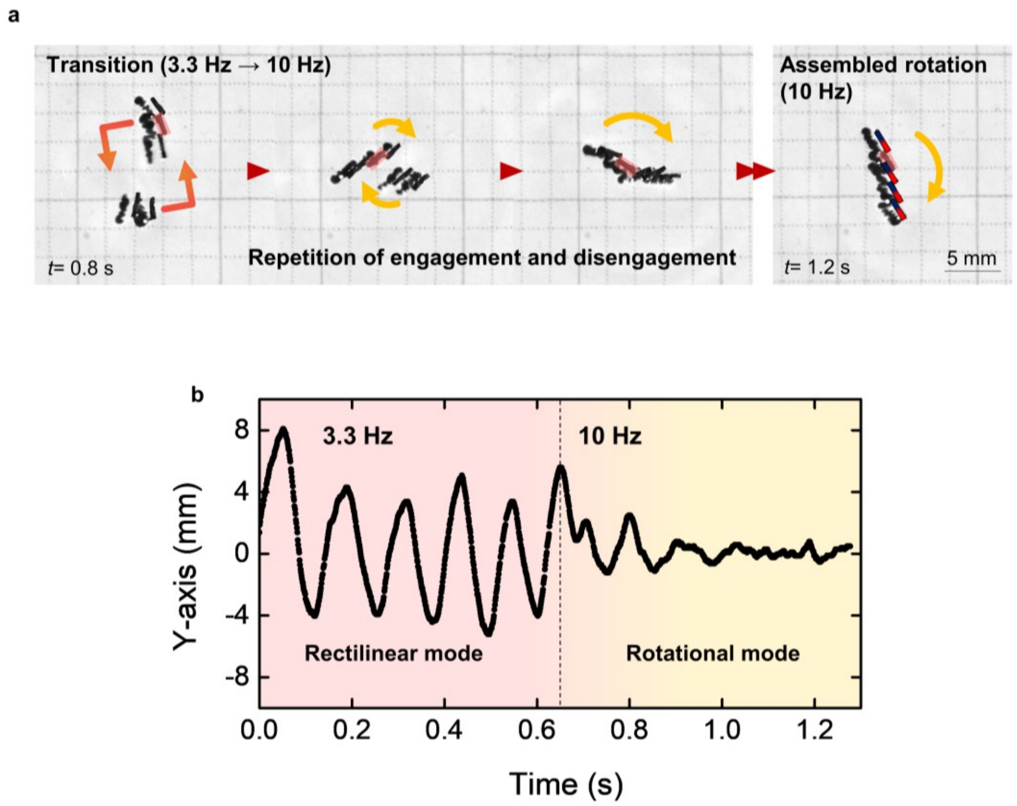

Supplementary Fig. 19 Adaptable magnetic organization of collectively swimming robots. (a) Time-lapse images and (b) trajectory of the red-colored CNTY robot upon manipulation of five 30 vol.% CNTY robots. Multiple robots performed assembled rectilinear translational swimming at 3.3 Hz. Increasing the magnetic frequency to 10 Hz resulted in reorganization of the modular CNTY robots for assembled rotational swimming. The CNTY robots were repeatedly engaged and disengaged in order that the opposing magnetic dipoles were encountered. Then the CNTY robots articulated magnetically to form the chain-like assembly.

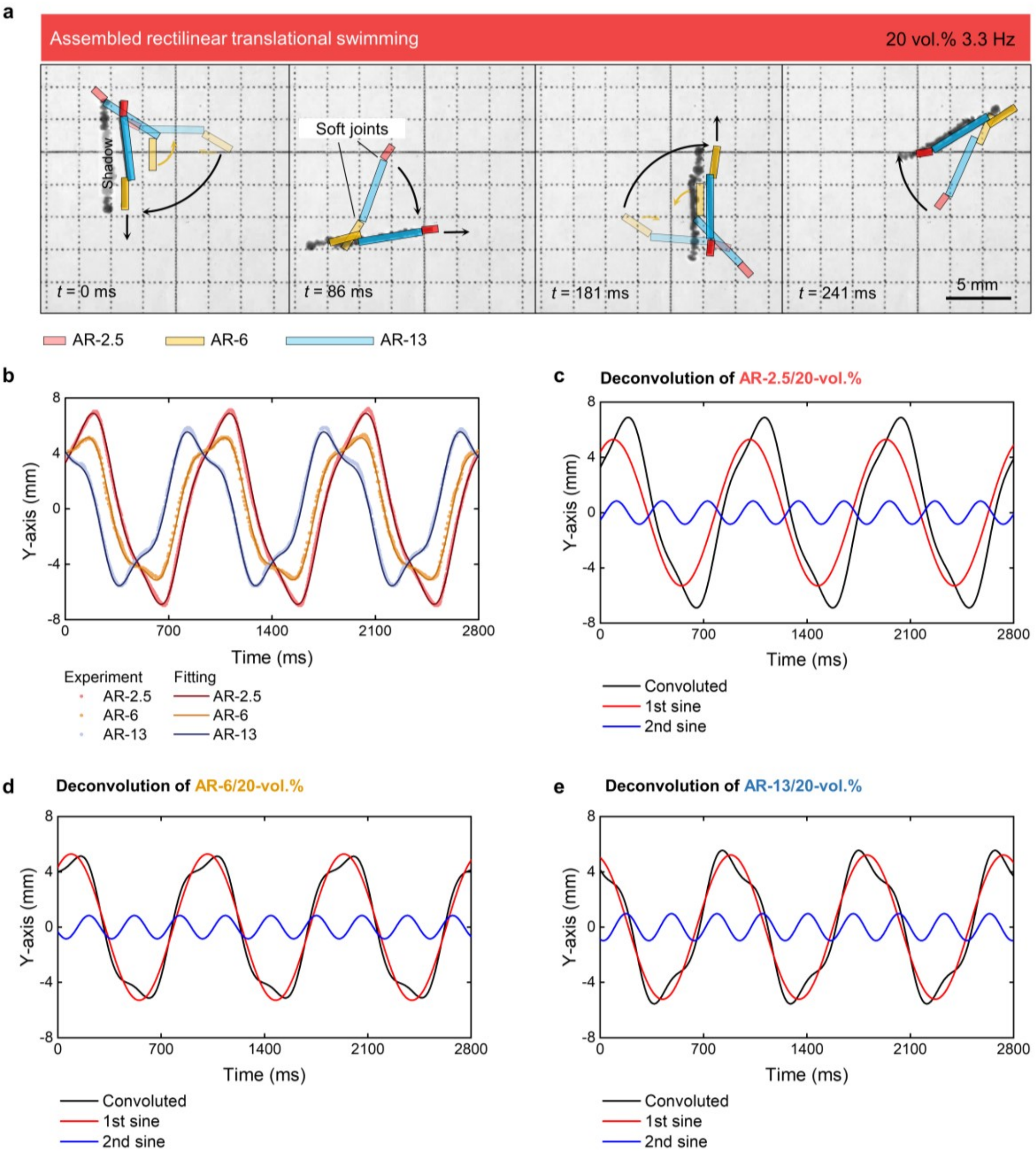

Supplementary Fig. 20 Assembled rectilinear translational swimming of multiple CNTY robots. Representative assembled rectilinear translational swimming of AR-2.5/20-vol.%, AR-6/20-vol.%, and AR-13/20-vol.% robots at a magnetic frequency of 3.3 Hz. (a) Time-lapse images. (b) *y*-axis coordinates of the three robots as a function of time. Deconvoluted sinusoidal waves of the (c) AR-2.5/20-vol.% robot (d) AR-6/20-vol.% robot, and (e) AR-13/20-vol.% robot affected by the magnetically interacting dynamic joints.

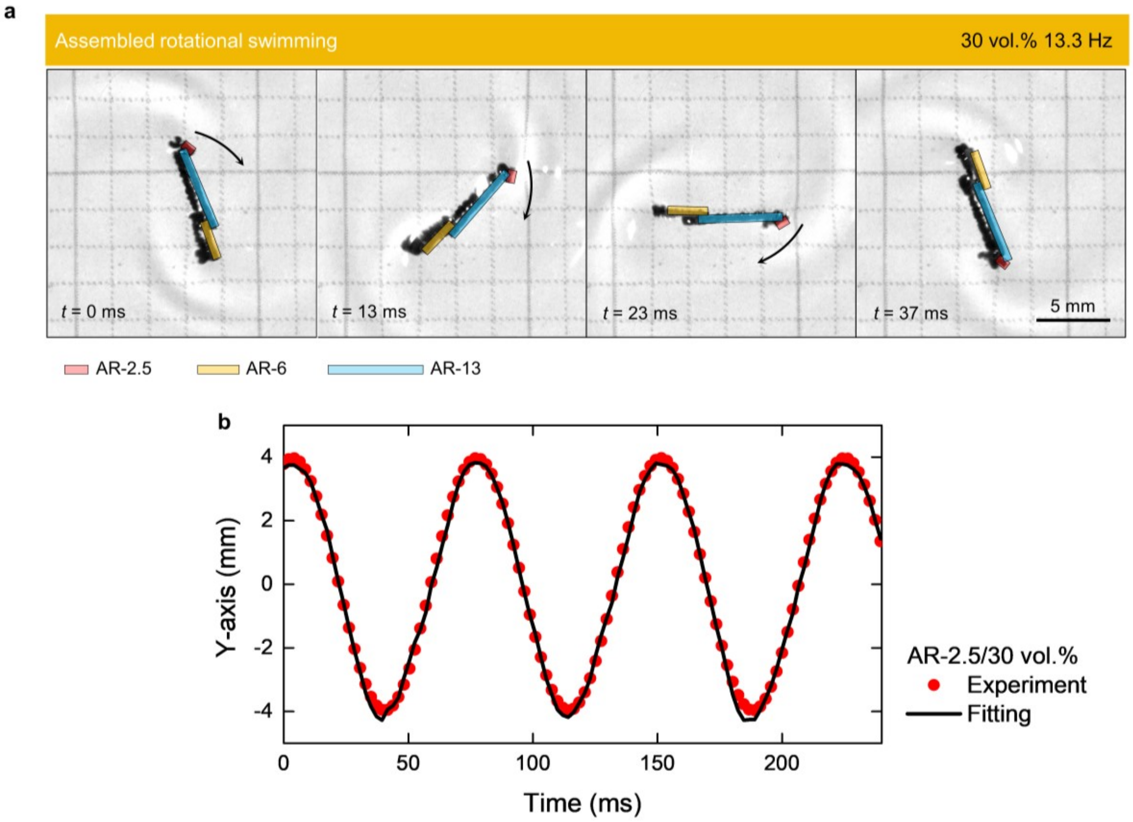

Supplementary Fig. 21 Assembled rotational swimming of multiple CNTY robots. Representative assembled rotational swimming of the AR-2.5/30-vol.%, AR-6/30-vol.%, and AR-13/30-vol.% robots at a magnetic frequency of 13.3 Hz. (a) Time-lapse images. (b) *y*-axis coordinates of the AR-2.5/30-vol.% robot as a function of time. The assembled rotational swimming data were fitted using a sinusoidal waveform of the *y*-axis.

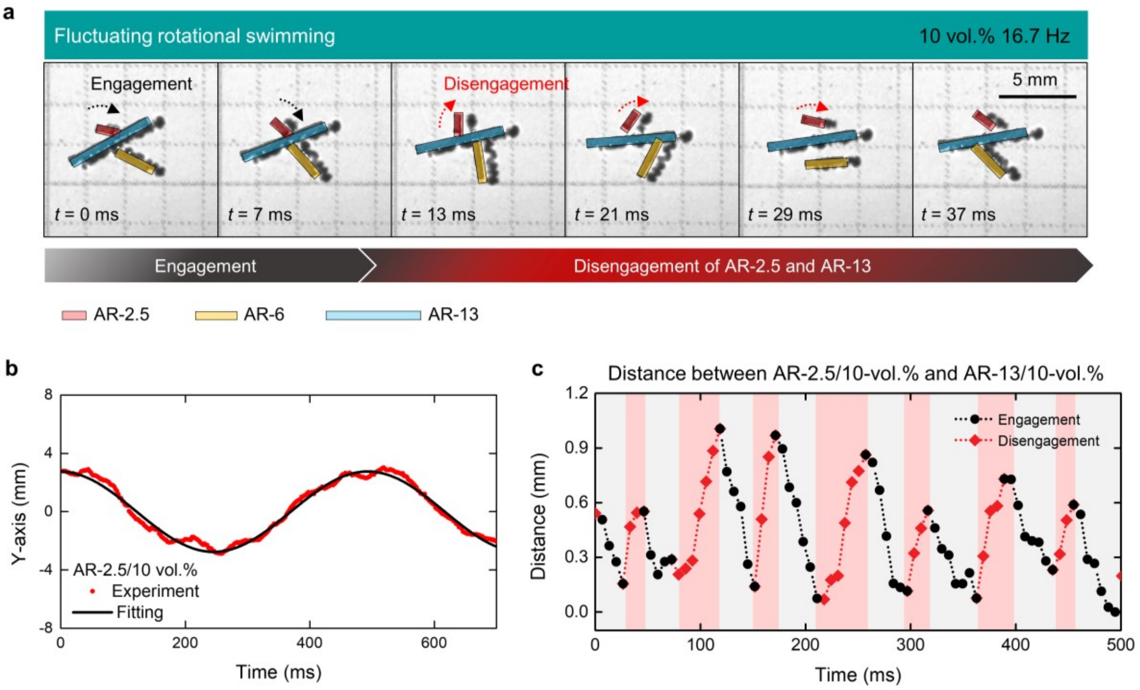

Supplementary Fig. 22 Fluctuating rotational swimming of multiple CNTY robots. Representative fluctuating rotational swimming of the AR-2.5/10-vol.%, AR-6/10-vol.%, and AR-13/10-vol.% robots at a magnetic frequency of 16.7 Hz. (a) Time-lapse images showing engagement and disengagement during the fluctuating rotational swimming mode. (b) Trajectory of fluctuating rotational swimming of the AR-2.5/10-vol.% robot fitted using a sinusoidal waveform of the *y*-axis. (c) Fluctuation of the distance between the AR-2.5/10-vol.% and AR-13/10-vol.% robots that was modulated by engagement and disengagement, as shown in Fig. 3c.

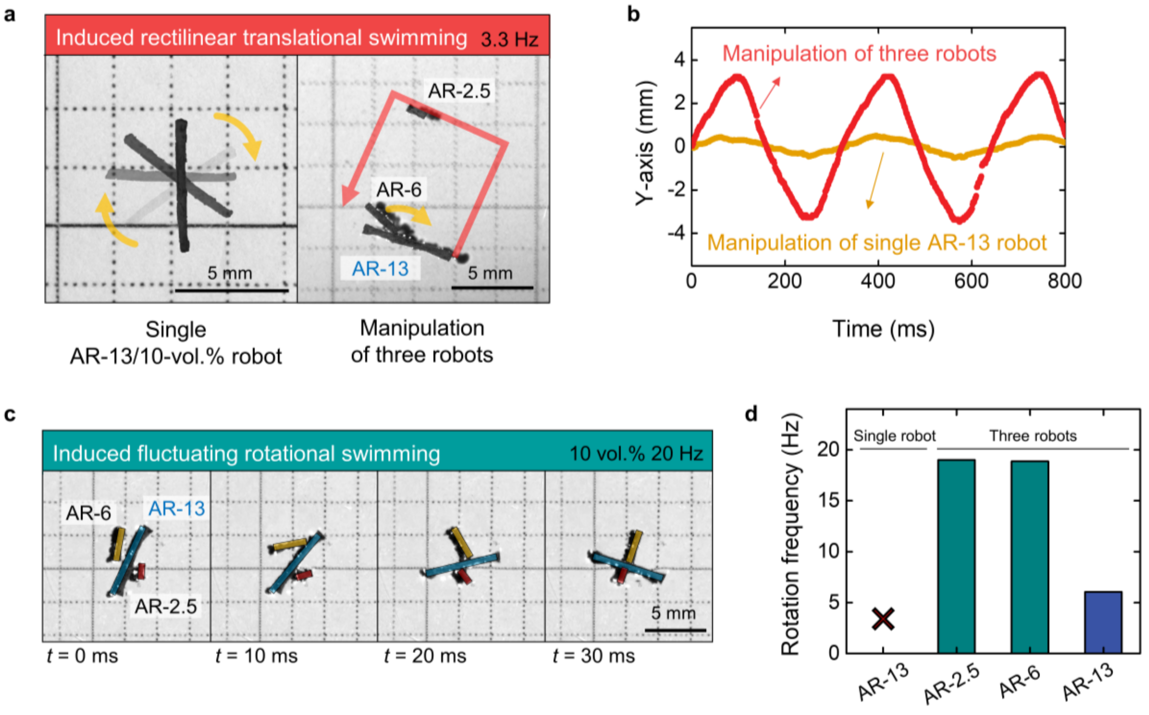

Supplementary Fig. 23 Enhanced motility through collective swimming. Manipulation of three (AR-2.5/10-vol.%, AR-6/10-vol.%, and AR-13/10-vol.%) CNTY robots. (a), (b) Induced rectilinear translational swimming of the AR-13/10-vol.% robot through collective swimming at a magnetic frequency of 3.3 Hz, confirmed by (a) overlaid images and (b) the *y*-axis trajectory. (c), (d) Induced fluctuating rotational swimming of the AR-13/10-vol.% robot through collective swimming at a magnetic frequency of 20 Hz, confirmed by (c) time-lapse images and (d) rotation frequency analysis. The single AR-13/10-vol.% robot was nonmotile at 20 Hz.

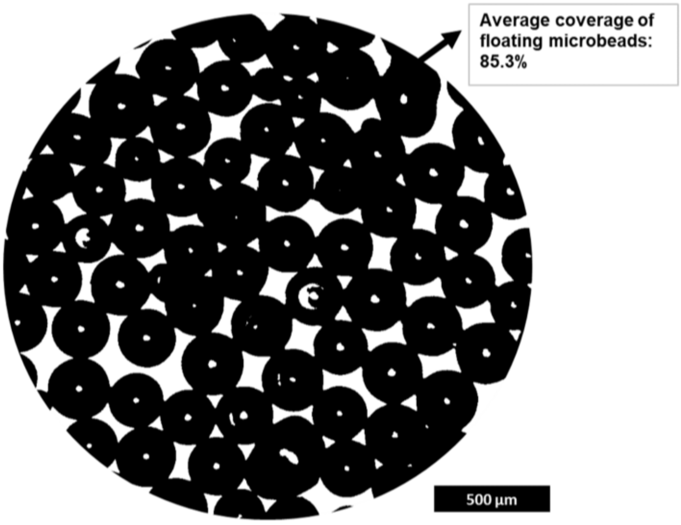

Supplementary Fig. 24. Binary image of floating microbeads. Black and white areas represented microbeads and non-coverage areas, respectively. The average coverage of floating microbeads was 85.3%. As light reflection and multi-focus issue of spheres hindered the image processing, the center of the microbead areas (white area) was excluded from the calculation of coverage area of microbead.

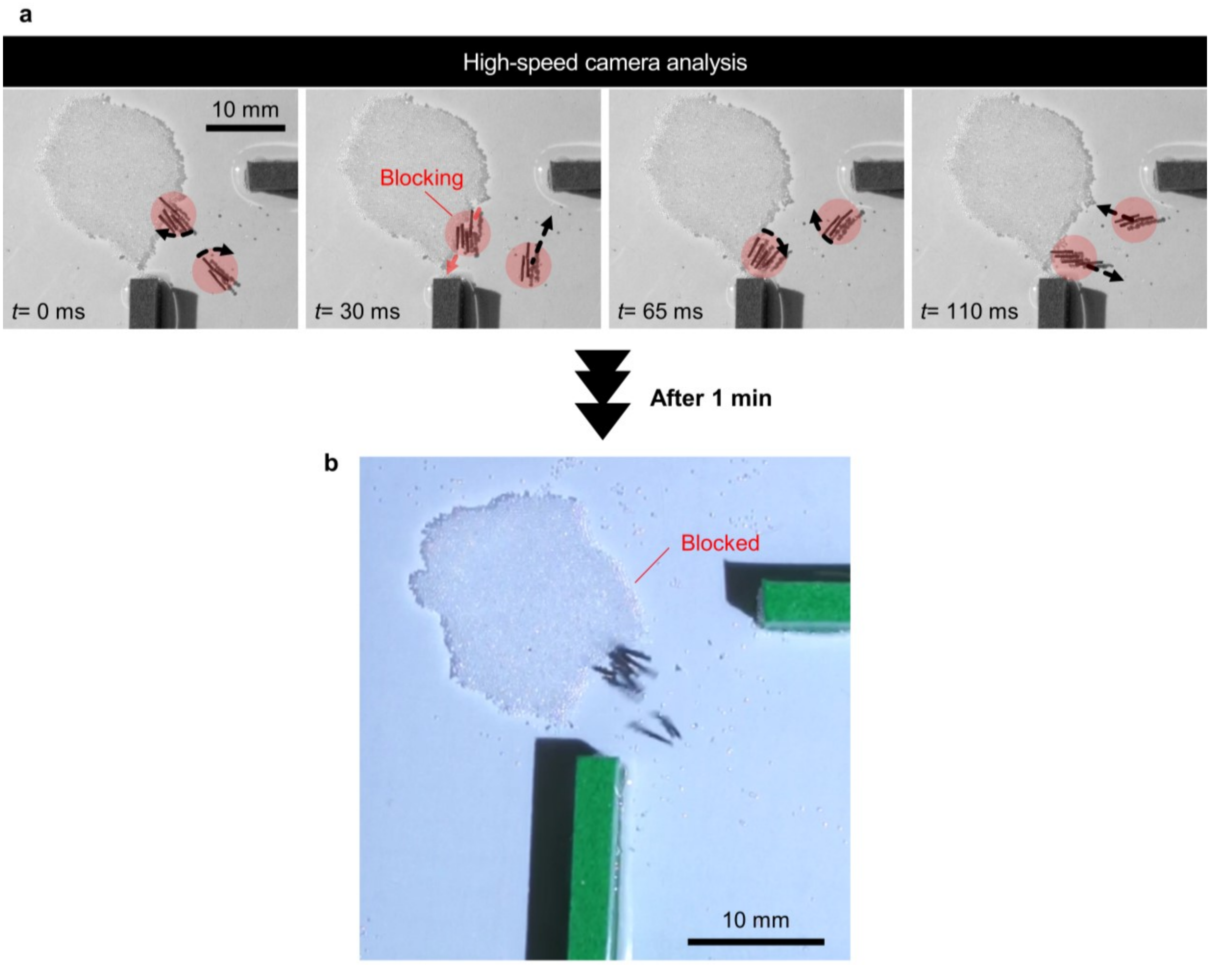

Supplementary Fig. 25 Physically blocked microbeads by assembled rectilinear translational swimming of the CNTY robots. Seven 10 vol.% CNTY robots were actuated at magnetic frequency of 3.3 Hz. The CNTY robots were located at the entrance of the divided space. (a) Physical blockage of the floating microbeads, confirmed by the high-speed camera. After the CNTY robots rotated at the vertex of the trajectory, the CNTY robots initiated rectilinear translational swimming, which could block the microbeads from entering. The agility of the swimming motion contributed to the physical blockage. (b) Prevented the influx of floating microbead over the course of 1 min, confirmed by digital camera.

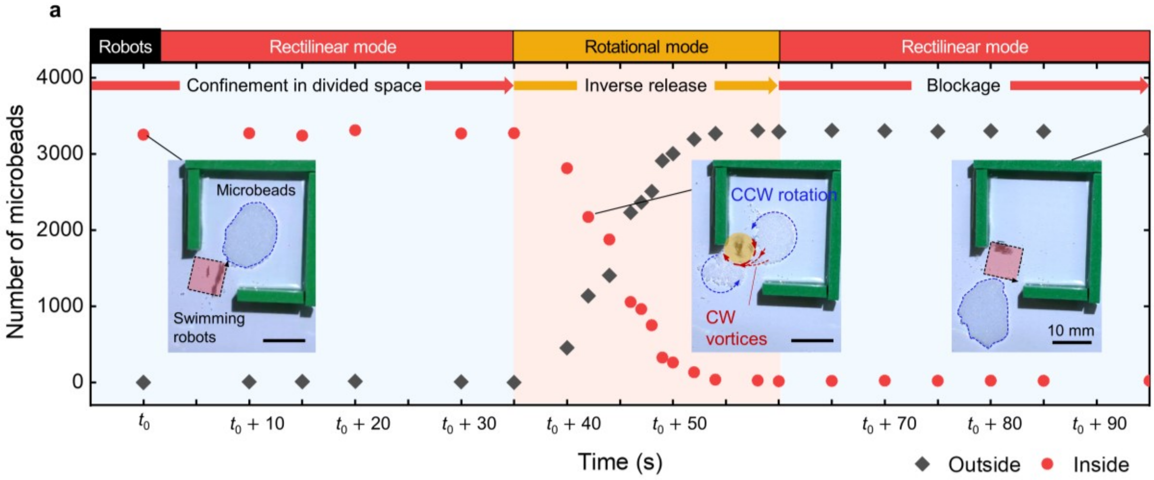

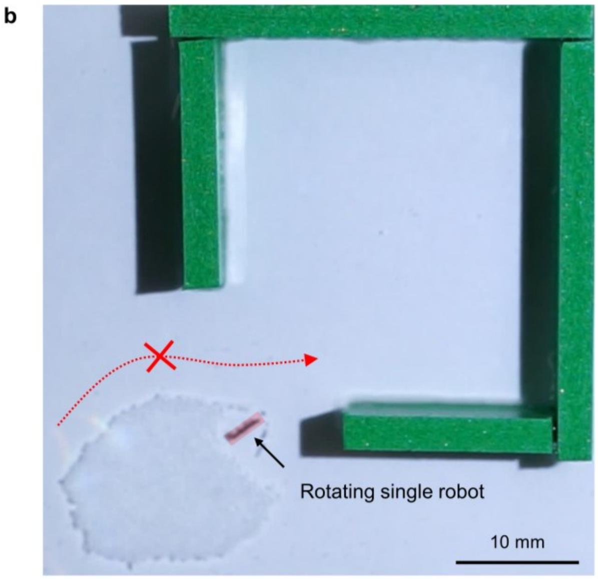

Supplementary Fig. 26 Release of floating microbeads by collective swimming of 10 vol.% CNTY robots. (a) Confinement, release, and blockage of 3,350 microbeads through appropriate swimming modes of seven 10 vol.% CNTY robots. The modular robots performed assembled rectilinear swimming at 3.3 Hz or assembled rotational swimming at 6.7 Hz. (b) Image indicating the incapability of a single AR-6/10-vol.% CNTY robot to transport microbeads above water.

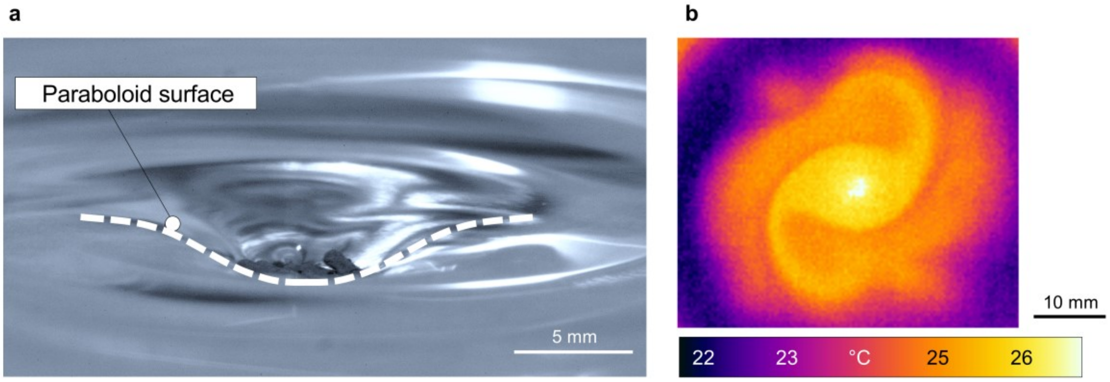

Supplementary Fig. 27 Vortex generation by collective swimming of 30 vol.% CNTY robots. Seven 30 vol.% CNTY robots functioning in the assembled rotational swimming mode, resulting in (a) a paraboloid water surface and (b) a large-magnitude CW vortex, which were confirmed using the high-speed camera and a forward-looking infrared (FLIR) imager, respectively. The magnetic frequency in (a) and (b) was 13.3 Hz and 10 Hz, respectively. The CW vortex was generated in the rotational modes (rotational swimming and fluctuating rotational swimming) from 10 to 20 Hz, as shown in Fig. 5b. A higher magnetic frequency generated a more significant vortex owing to the increased angular velocity of the vortex.

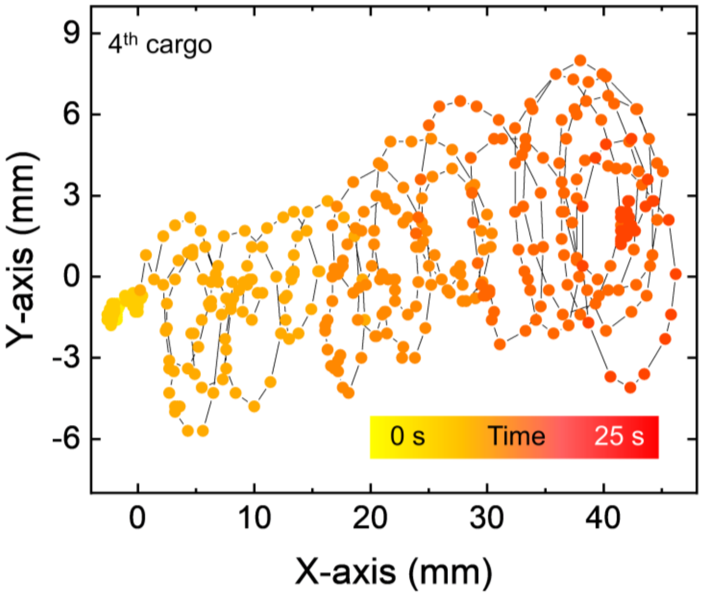

Supplementary Fig. 28 Transportation of semi-submerged cargo. Trajectory of the fourth semi-submerged cargo shown in Fig. 5c.

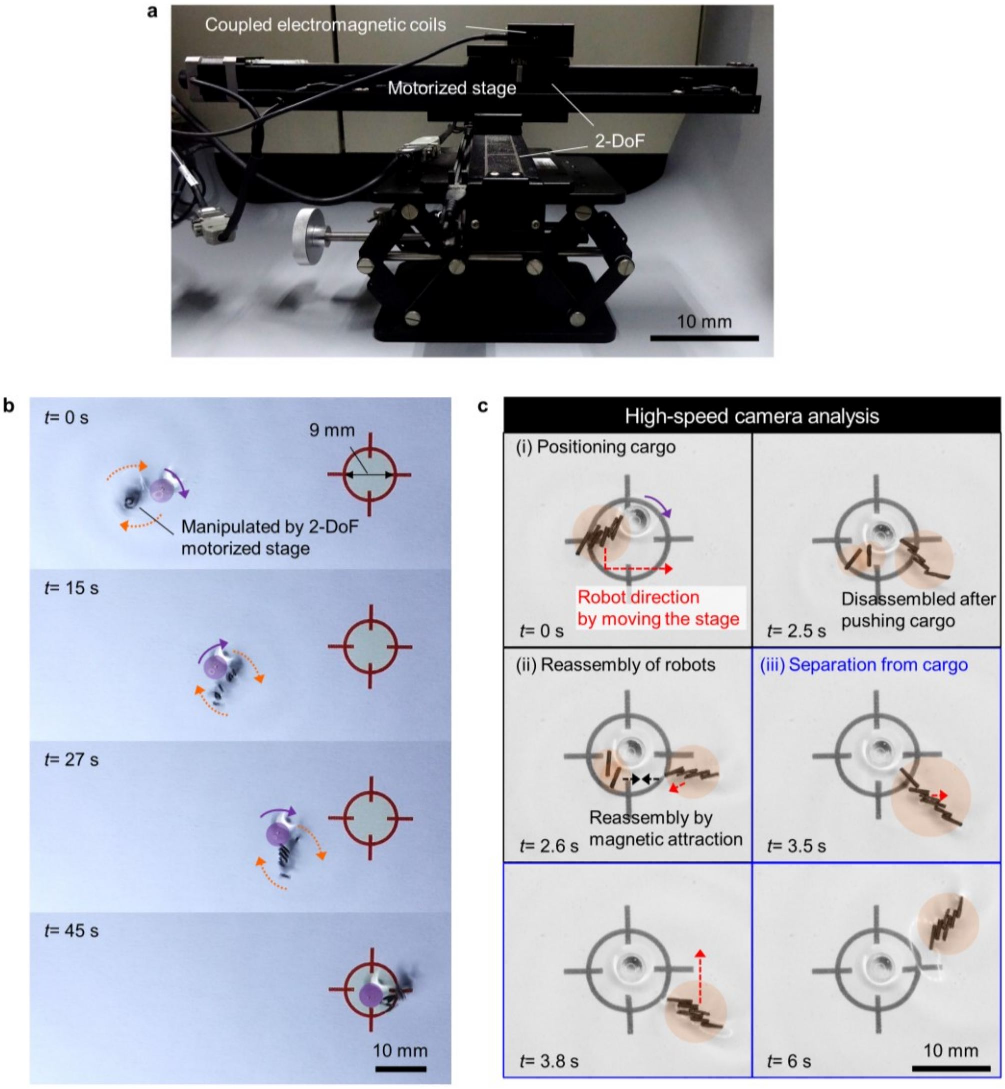

Supplementary Fig. 29 Transportation of semi-submerged spherical cargo by manipulating the motorized stage. (a) Motorized stage having two degrees of freedom (2-DoF) coupled with the electromagnetic coils. (b) Delicate positional control of seven 30 vol.% CNTY robots in order to transport the spherical cargo by manipulating the location of the motorized stage. Diameter and weight of the cargo were 4 mm and 52 mg, respectively. The magnetic frequency was 13.3 Hz. (c) Cargo placement process, confirmed by high-speed camera. When six 30 vol.% CNTY robots moved through assembled rotational swimming, they were able to push the cargo by elastic energy transfer. As a result, cargo delivery was successful. Although the CNTY robots disassembled, they instantaneously reassembled by magnetic attractive force among the robots and could swim through assembled rotational swimming. Since CNTY robots spontaneously assembled even after disassembly, location control of multiple CNTY robots was achieved with ease. By simply moving the CNTY robots away from the cargo before pushing the cargo again, all robots could be separated immediately from the cargo. The positioning and separating process of the cargo were implemented solely through manipulation of the motorized stage location without varying the magnetic frequency of the magnetic field.

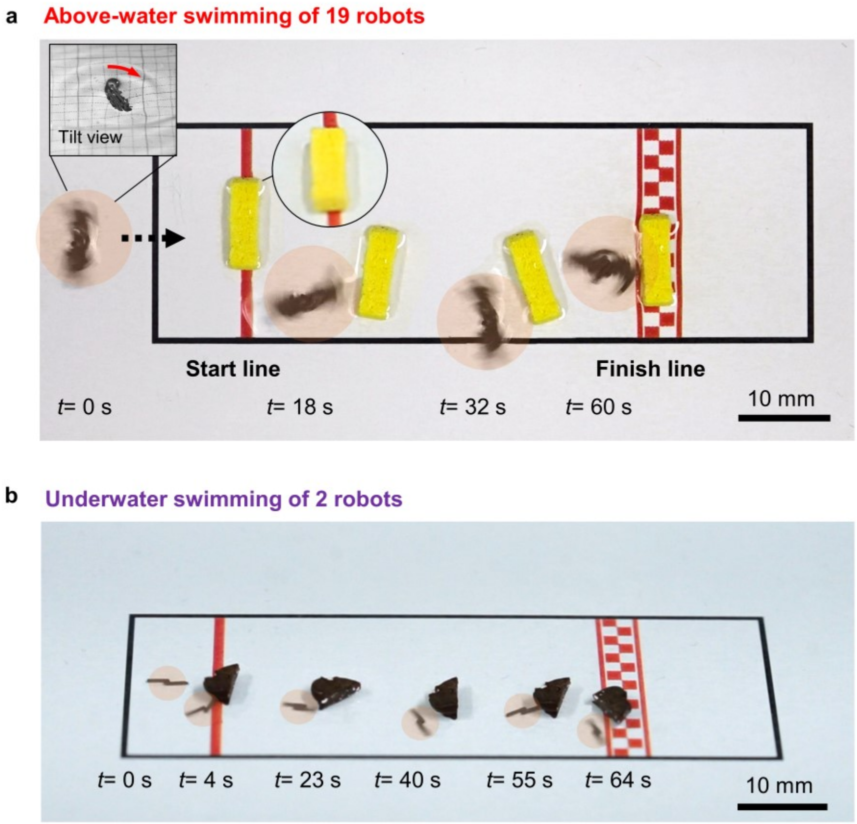

Supplementary Fig. 30 Transportation of cargos with sliding resistance by above-water swimming and underwater swimming. Multiple 30 vol.% CNTY robots were manipulated by moving the electromagnetic coils coupled with the motorized stage having two degrees of freedom (2-DoF). (a) Transportation of semi-submerged cuboid cargo with a weight of 42 mg by above-water swimming of 19 CNTY robots. The increased number of CNTY robots resulted in a larger magnitude of vortex due to the larger hydrodynamic volume of the magnetic modular assembly. Therefore, the 19 CNTY robots with assembled rotational swimming transported cuboid cargo with sliding resistance. (b) Transportation of submerged asymmetric cargo with a weight of 39 mg by underwater swimming of two CNTY robots. The underwater swimming was proceeded after the robots were intentionally submerged in water. When the CNTY robots were pressed to the bottom of the water container, the robots sank immediately and remained submerged. When the sunken CNTY robots were relocated on the surface of water, they performed agile above-water swimming without submerging due to water's natural surface tension. The magnetic frequency was 8.3 Hz in (a) and (b).

Supplementary Note 1. Rheological and magnetization properties of ternary nanocomposite

The complex viscosities of the PDMS–iron-particle mixtures were measured using a parallel-plate rheometer (MCR302, Anton Paar) with a plate gap of 5 mm. The experiments were conducted at 23.8 °C and angular frequencies of 1–100 rad s^{-1}. The complex viscosity was calculated at an angular frequency of 3 rad s^{-1}.

At low angular frequencies, the storage and loss moduli show slopes of 2 and 1 against the shear rate, respectively, which is a typical rheological characteristic of Newtonian liquids (Supplementary Fig. 8–10). The complex viscosity of the PDMS prepolymer was measured to be 18 Pa·s at an angular frequency of 3 rad s^{-1}. When 1 and 2 vol.% magnetic particles were dispersed in the PDMS prepolymer, a magnetic composite layer did not appear owing to the insignificant difference in the values of complex viscosity compared to that of the PDMS prepolymer. The viscosity increased to 33, 90, and 371 Pa·s at iron particle loadings of 5, 10, and 20 vol.%, resulting in 10-, 14-, and 21-μm-thick magnetic composite layers on the CNTY after thermal curing, respectively; consequently, M_s was estimated to be 40, 69, and 118 emu g^{-1}, respectively (Supplementary Fig. 11). Randomly distributed spherical particles typically exhibit rheological percolation thresholds between 20 and 30 vol.%. Predictably, the viscosity and coating thickness drastically increased to 897 Pa·s and 60 μm, respectively, at an iron particle loading of 30 vol.%, with an M_s of 152 emu g^{-1} being obtained after the polymerization. Compared to the M_s value of bulk iron particles (237 emu g^{-1}), the large M_s of the composites prevented the aggregation of iron particles by the PDMS binder. In the case of the iron particle concentration of 40 vol.%, the highly viscous mixture of the prepolymer composite could not be drained because of its high complex viscosity (2,056 Pa·s), leading to non-uniform coating onto the CNTY surface. The iron particle concentration of 5 vol.% was excluded because of the inability of the resulting robot to swim.

Supplementary Note 2. Swimming mode analysis of a single robot

Rectilinear translational swimming and rotational swimming involve x-axis and y-axis coordinates, as expressed by the following equations representing the Lissajous–Bowditch curve:

$$x(t) = a_0 \sin(\omega t), \quad (1)$$

$$y(t) = b_0 \sin(\omega t + \delta), \quad (2)$$

where $x(t)$ and $y(t)$ are functions of time (t), δ denotes the phase difference, a_0 and b_0 are constants for the amplitude, and ω is the frequency of the trajectory. Each periodic wave, $f(t)$, of the coordinates was fitted to a Fourier series, as follows:

$$f(t) = \sum_{n=1}^{n} c_n \sin(n\omega t) + d_n \cos(n\omega t), \quad (3)$$

where the upper limit, n, was set to 3 in this study to account for the third harmonics, and c_n and d_n are constants. As shown in Supplementary Fig. 14, the ideal counterclockwise (CCW) rectilinear translational swimming exhibited a trapezoidal wave, in which the phase difference had a positive value of 1.57 rad. In contrast, the ideal centroid–vertex distance during clockwise (CW) rotational swimming was zero without a phase difference.

Because the trapezoidal waveform in CCW rectilinear translational swimming contained odd Fourier components, the swimming modes were experimentally elucidated by confirming the phase difference in the deconvoluted odd harmonics (Supplementary Fig. 15). A representative rectilinear translational trajectory was demonstrated by the AR-2.5/20-vol.% robot at a magnetic frequency of 6.7 Hz. The CNTY robot exhibited a noticeable signal in the fundamental component of the odd harmonics with a positive phase difference of 1.59 rad. At the third harmonic, a weak signal was observed with a negative phase difference of −1.72 rad. The positive phase difference with a dominant amplitude in the fundamental component indicates CCW rectilinear translational swimming. With respect to rotational swimming, the AR-2.5/10-vol.% CNTY robot exhibited insignificant signals in the harmonics without a phase delay at a similar magnetic frequency.

Supplementary references

1. Hu, D. L., Chan, B. & Bush, J. W. M. The hydrodynamics of water strider locomotion. *Nature* **424,** 663–666 (2003).

2. Aigeldinger, T. & Fish, F. Hydroplaning by ducklings: overcoming limitations to swimming at the water surface. *J. Exp. Biol.* **198,** 1567–1574 (1995).

3. Hsieh, S. T. & Lauder, G. V. Running on water: Three-dimensional force generation by basilisk lizards. *Proc. Natl. Acad. Sci.* **101,** 16784–16788 (2004).

4. Rubinoff, I., Graham, J. B. & Motta, J. Diving of the sea snake Pelamis platurus in the Gulf of Panamá. *Mar. Biol.* **91,** 181–191 (1986).

5. Ghanbari, A. Bioinspired reorientation strategies for application in micro/nanorobotic control. *J. Micro-Bio Robot.* **16,** 173–197 (2020).

6. Rezai, P., Siddiqui, A., Selvaganapathy, P. R. & Gupta, B. P. Electrotaxis of Caenorhabditis elegans in a microfluidic environment. *Lab Chip* **10,** 220–226 (2010).

7. Enright, J. T. Copepods in a hurry: Sustained high-speed upward migration. *Limnol. Oceanogr.* **22,** 118–125 (1977).

8. Arnott, S. A., Neil, D. M. & Ansell, A. D. Tail-flip mechanism and size-dependent kinematics of escape swimming in the brown shrimp Crangon crangon. *J. Exp. Biol.* **201,** 1771–1784 (1998).

9. Perry, M. J., Tait, J., Hu, J., White, S. C. & Medler, S. Skeletal muscle fiber types in the ghost crab, Ocypode quadrata: implications for running performance. *J. Exp. Biol.* **212,** 673–683 (2009).

10. Lang, T. G. & Norris, K. S. Swimming speed of a Pacific bottlenose porpoise. *Science* **151,** 588–590 (1966).

11. Fish, F. E. Comparative kinematics and hydrodynamics of odontocete cetaceans: morphological and ecological correlates with swimming performance. *J. Exp. Biol.* **201,** 2867–2877 (1998).

12. Bryan, M. T. *et al.* Microscale magneto-elastic composite swimmers at the air-water and water-solid interfaces under a uniaxial field. *Phys. Rev. Appl.* **11,** 044019 (2019).

13. He, Y. *et al.* Design, analysis and experiments of a magnetic microrobot capable of locomotion and manipulation at water surfaces. *J. Micromechanics Microengineering* **29,** 025010 (2019).

14. Won, S., Kim, S., Park, J. E., Jeon, J. & Wie, J. J. On-demand orbital maneuver of multiple soft

robots via hierarchical magnetomotility. *Nat. Commun.* **10,** 4751 (2019).

15. Lum, G. Z. *et al.* Shape-programmable magnetic soft matter. *Proc. Natl. Acad. Sci. U. S. A.* **113,** E6007–E6015 (2016).

16. Yu, S. *et al.* Self-propelled janus microdimer swimmers under a rotating magnetic field. *Nanomaterials* **9,** 1672 (2019).

17. Xie, M. *et al.* Bioinspired soft microrobots with precise magneto-collective control for microvascular thrombolysis. *Adv. Mater.* **32,** 2000366 (2020).

18. Ji, S., Li, X., Chen, Q., Lv, P. & Duan, H. Enhanced locomotion of shape morphing microrobots by surface coating. *Adv. Intell. Syst.* **3,** 2000270 (2021).

19. Won, S., Je, H., Kim, S. & Wie, J. J. Agile underwater swimming of magnetic polymeric microrobots in viscous solutions. *Adv. Intell. Syst.* **4,** 2100269 (2022).

20. Bi, C., Guix, M., Johnson, B. V., Jing, W. & Cappelleri, D. J. Design of microscale magnetic tumbling robots for locomotion in multiple environments and complex terrains. *Micromachines* **9,** 68 (2018).

21. Chen, W. *et al.* Triple-configurational magnetic robot for targeted drug delivery and sustained release. *ACS Appl. Mater. Interfaces* **13,** 45315–45324 (2021).

22. Hunter, E. E., Brink, E. W., Steager, E. B. & Kumar, V. 3D micromolding of small-scale biological robots. in *2018 International Conference on Manipulation, Automation and Robotics at Small Scales (MARSS)* 1–6 (IEEE, 2018). doi:10.1109/MARSS.2018.8481196

23. Li, C. *et al.* Fast and programmable locomotion of hydrogel-metal hybrids under light and magnetic fields. *Sci. Robot.* **5,** eabb9822 (2020).

24. Choi, J. *et al.* Flexible and robust thermoelectric generators based on all-carbon nanotube yarn without metal electrodes. *ACS Nano* **11,** 7608–7614 (2017).

25. Lee, J. *et al.* Direct spinning and densification method for high-performance carbon nanotube fibers. *Nat. Commun.* **10,** 2962 (2019).
