# Supplementary material for: Multimodal collective swimming of magnetically articulated modular nanocomposite robots
Source: Nat Commun. 2022 Nov 8;13:6750. doi: 10.1038/s41467-022-34430-2 (PMC9643480; doi:10.1038/s41467-022-34430-2)
Supplement: Supplementary file 3 — Description of Additional Supplementary Files [file 41467_2022_34430_MOESM3_ESM.pdf]

Description of Additional Supplementary Files

Supplementary Movie 1. Bimodal swimming of CNTY robots.

Supplementary Movie 2. Swimming mode analysis of a single CNTY robot.

Supplementary Movie 3. Adaptable magnetic organization of multiple modular robots.

Supplementary Movie 4. Simultaneous swimming of multiple modular robots.

Supplementary Movie 5. Vortex control for transporting > 3,000 floating microbeads in divided space.

Supplementary Movie 6. Generation of a large-magnitude vortex.

Supplementary Movie 7. Chiral inversion in the vortex.

Supplementary Movie 8. Transportation of semi-submerged cargo.

Supplementary Movie 9. Improvement of transportation precision using motorized stage.

Supplementary Movie 10. Collection of > 4,000 floating microplastics using motorized stage.
